# Endoscopic transpapillary gallbladder drainage using a novel sphincterotome

**DOI:** 10.1111/den.14943

**Published:** 2024-10-22

**Authors:** Takuya Ishikawa, Ryohei Kumano, Hiroki Kawashima

**Affiliations:** ^1^ Nagoya University Graduate School of Medicine Aichi Japan

## Abstract

Watch a video of this article.

## BRIEF EXPLANATION

**Figure 1:**
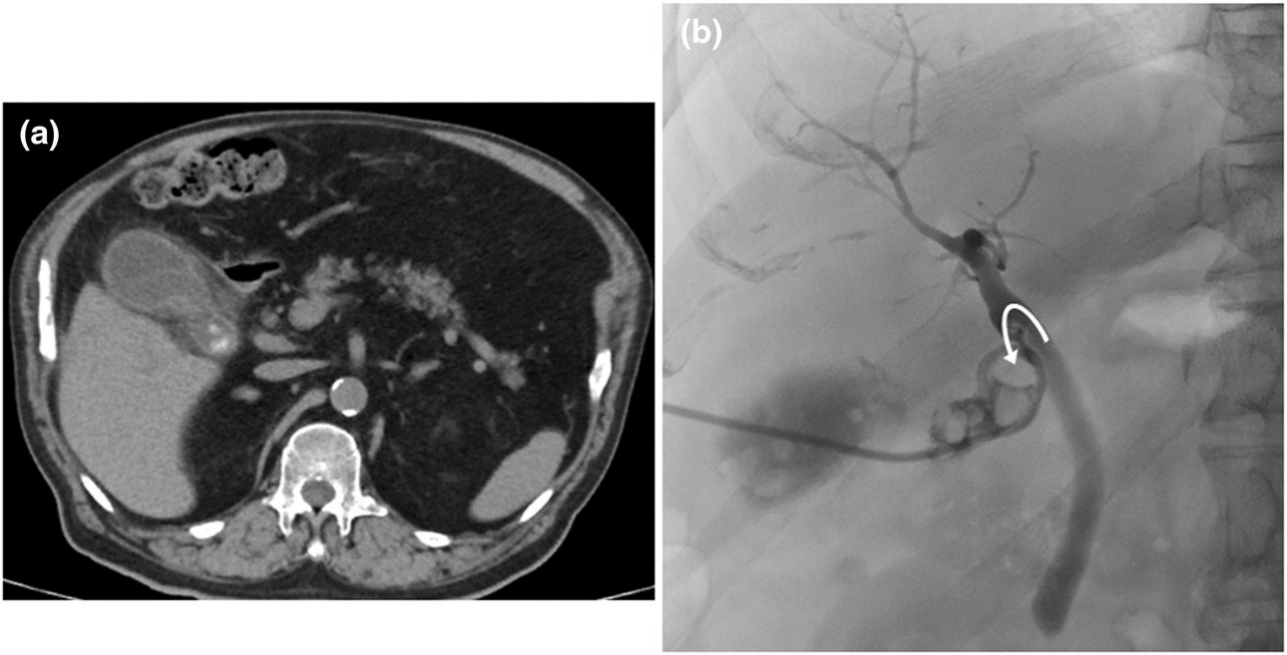
(a) Computed tomography scan showing cholecystitis with stones in the neck of the gallbladder. (b) Cholangiography during percutaneous transhepatic gallbladder drainage showing the cystic duct branching to the caudal side (arrow).

**Figure 2:**
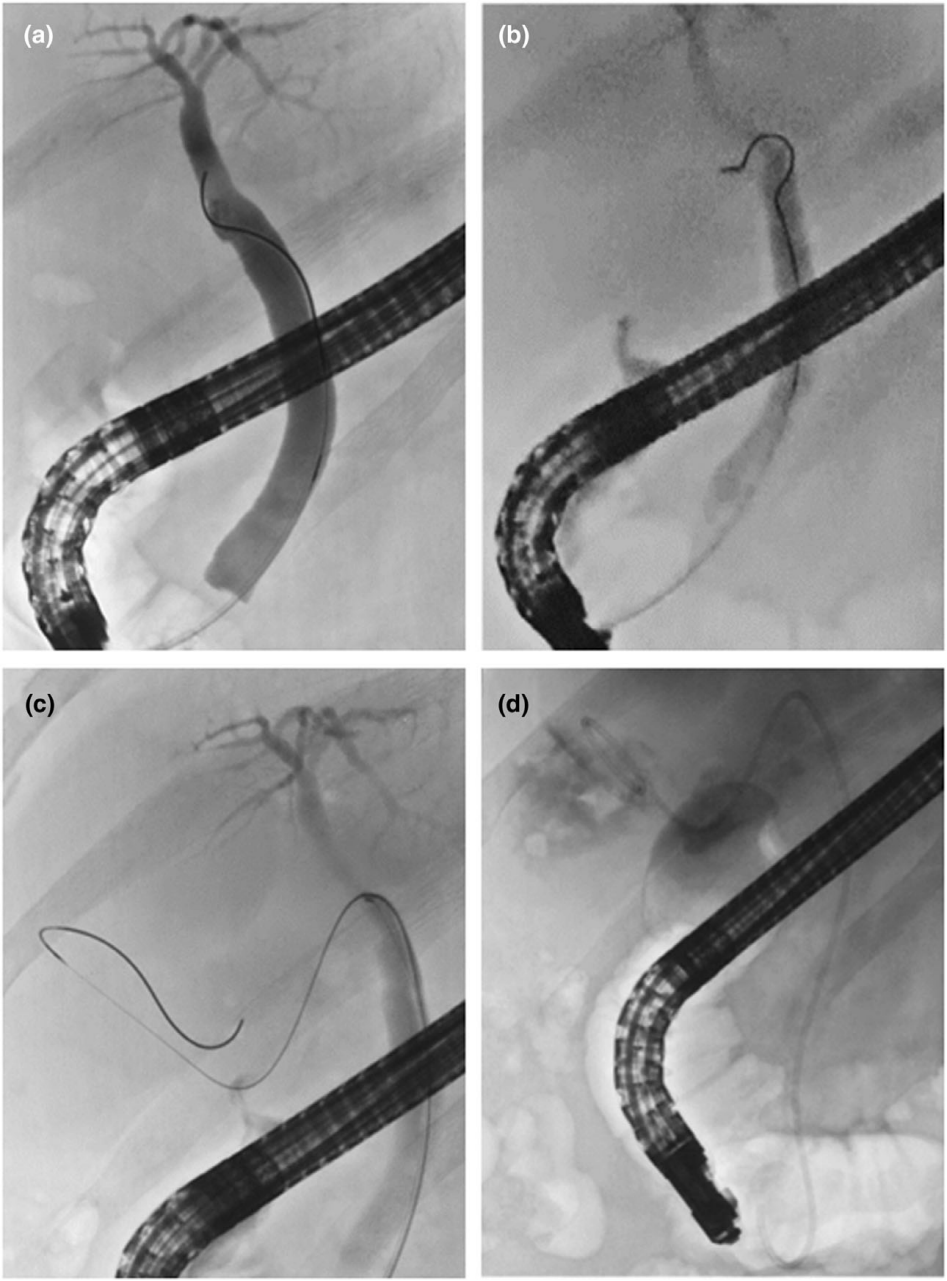
Images during endoscopic transpapillary gallbladder drainage. (a) Catheterization of the cystic duct showing that it was difficult to insert the guidewire into the cystic duct. (b) Bending and rotating the tip of a novel sphincterotome inside the bile duct to adjust the direction toward the cystic duct. (c) Successful passage of the guidewire into the gallbladder. (d) Plastic stent placement in the gallbladder.

Endoscopic transpapillary gallbladder drainage (ETGBD) has been reported as an option for patients for whom percutaneous transhepatic gallbladder drainage (PTGBD) or cholecystectomy is not indicated.^1^ Compared with PTGBD, ETGBD is expected to have a lower patient burden and a shorter hospital stay because of internal drainage. However, ETGBD is technically challenging, and some cases are difficult to treat. In particular, it has been reported that guidewire or catheter manipulation is difficult when the cystic duct branches are to the caudal side because of steep angulation.^2^ We report a case of successful ETGBD using a novel sphincterotome (ENGETSU; Kaneka Medix Corporation, Osaka, Japan) (Video S1). The patient suffered from recurrent cholecystitis, and ETGBD was planned, but previous cholangiography during PTGBD revealed the cystic duct branching to the caudal side (Fig. 1). The ENGETSU is a newly developed rotatable sphincterotome with a wide range of motion that enables easy control of the tip compared to the conventional sphincterotome, which only has bending ability. A normal straight tip catheter was initially used to access the cystic duct, but it was unsuccessful because of the steep angle between the common bile duct and the cystic duct(Fig. 2a). A novel sphincterotome was then used to adjust the direction toward the cystic duct by bending and rotating the tip (Fig. 2b), and the guidewire was successfully advanced into the gallbladder (Fig. 2c). A 5F plastic stent dedicated for gallbladder drainage (IYO stent; Gadelius Medical K.K., Tokyo, Japan)^3^ was placed (Fig. 2d), and purulent bile was drained immediately after stent placement. In this procedure, the main operator was a trainee, whereas the assistant was an expert, and one assistant could manipulate both the tip of the sphincterotome and guidewire. In conclusion, the novel sphincterotome, with its rotatable tip and wide range of motion, is useful in difficult ETGBD cases in which the cystic duct branches to the caudal side (Figs 1,2).

Authors declare no conflict of interest for this article.

## Supporting information


**Video S1** Endoscopic transpapillary gallbladder drainage using a novel sphincterotome.
